# The Antioxidant Moiety of MitoQ Imparts Minimal Metabolic Effects in Adipose Tissue of High Fat Fed Mice

**DOI:** 10.3389/fphys.2019.00543

**Published:** 2019-05-08

**Authors:** Simon T. Bond, Jisu Kim, Anna C. Calkin, Brian G. Drew

**Affiliations:** ^1^Baker Heart and Diabetes Institute, Melbourne, VIC, Australia; ^2^Central Clinical School, Monash University, Melbourne, VIC, Australia

**Keywords:** mitoquinone, adipose tissue, ROS, antioxidant, mitochondria, obesity

## Abstract

Mitochondrial dysfunction is associated with a diverse array of diseases ranging from dystrophy and heart failure to obesity and hepatosteatosis. One of the major biochemical consequences of impaired mitochondrial function is an accumulation of mitochondrial superoxide, or reactive oxygen species (ROS). Excessive ROS can be detrimental to cellular health and is proposed to underpin many mitochondrial diseases. Accordingly, much research has been committed to understanding ways to therapeutically prevent and reduce ROS accumulation. In white adipose tissue (WAT), ROS is associated with obesity and its subsequent complications, and thus reducing mitochondrial ROS may represent a novel strategy for treating obesity related disorders. One therapeutic approach employed to reduce ROS abundance is the mitochondrial-targeted coenzyme Q (MitoQ), which enables mitochondrial specific delivery of a CoQ10 antioxidant via its triphenylphosphonium bromide (TPP+) cation. Indeed, MitoQ has been successfully shown to accumulate at the outer mitochondrial membrane and prevent ROS accumulation in several tissues *in vivo*; however, the specific effects of MitoQ on adipose tissue metabolism *in vivo* have not been studied. Here we demonstrate that mice fed high-fat diet with concomitant administration of MitoQ, exhibit minimal metabolic benefit in adipose tissue. We also demonstrate that both MitoQ and its control agent dTPP+ had significant and equivalent effects on whole-body metabolism, suggesting that the dTPP+ cation rather than the antioxidant moiety, was responsible for these changes. These findings have important implications for future studies using MitoQ and other TPP+ compounds.

## Introduction

Obesity and the metabolic syndrome (MS) are associated with a number of co-morbidities and have been established as a rapidly growing epidemic, particularly in the developed world (Han and Lean, 2016). It is now known that mitochondria play an important role in maintaining WAT health (Bournat and Brown, 2010; Kusminski et al., 2012), and one of the proposed pathological drivers of obesity and MS is dysfunction of mitochondria in white adipose tissue (WAT) (Attie and Scherer, 2009; Bournat and Brown, 2010). There are many factors that contribute to mitochondrial dysfunction. Indeed, reactive oxygen species (ROS) are elevated in adipose tissue in a number of metabolic disorders, including obesity and MS, and are thought to contribute to the progressive decline in WAT cellular health (Furukawa et al., 2004). Although the accumulation of ROS is traditionally viewed as being pathological, several studies have demonstrated ROS to be an important intracellular signaling molecule; however, ROS homeostasis and the mechanisms by which ROS affects cellular processes, remains incompletely understood. Previous studies have established that elevations in glucose and free fatty acids, often associated with obesity and MS, can promote the generation of ROS in adipocytes (Talior et al., 2003). In turn, ROS in adipocytes have been shown to regulate lipolysis, cell differentiation (Tormos et al., 2011; Krawczyk et al., 2012), cell signaling (Krawczyk et al., 2012), and cellular dysfunction (Furukawa et al., 2004).

A large proportion of ROS is generated by the mitochondrial electron transport chain (ETC), which produces superoxide via the reduction of oxygen (Murphy, 2009). Superoxide then undergoes dismutation by superoxide dismutase 1, 2, and 3 (SOD1, SOD2, and SOD3) to form the ROS, hydrogen peroxide (Rosen et al., 1993; Murphy, 2009). An accumulation of both superoxide and hydrogen peroxide causes oxidative damage, which can lead to cellular dysfunction, and death (Pandey and Rizvi, 2010), often via the formation of reactive lipid species formed by lipid peroxidation (Higdon et al., 2012).

**FIGURE 1:**
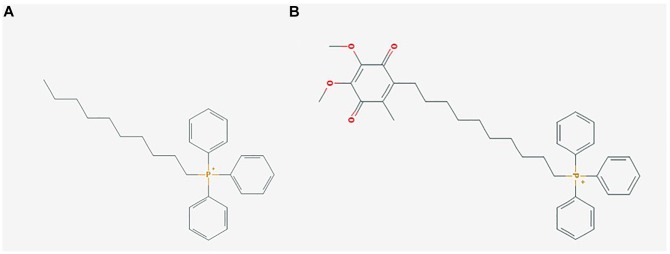
Mitochondrial-targeted coenzyme Q (MitoQ) is a mitochondrial targeting antioxidant with a ubiquinone moiety attached to a dTPP+ cation. **(A)** (1-Decyl)triphenylphosphonium bromide (dTPP+), a positively charged lipophilic cation consisting of a 10 carbon chain attached to a triphenylphosphonium bromide moiety, PubChem CID: 3084561; **(B)** Mitoquinone (MitoQ), consisting of the antioxidant ubiquinone, which is identical to the active antioxidant in Coenzyme Q10, attached to a dTPP+ cation, PubChem CID: 11388331. Images sourced from the PubChem Compound Database, National Center for Biotechnology Information (2019a,b).

Manipulating mitochondrial ROS has been an area of therapeutic interest for conditions such as obesity, MS and type 2 diabetes, albeit with little success. A mitochondrial specific antioxidant, mitoquinone (MitoQ), was designed by Kelso et al. (2001) as a potential therapeutic to target mitochondrial ROS. MitoQ is a derivative of coenzyme Q (Figure 1), which has a quinone antioxidant moiety linked to a saturated ten carbon alkyl chain with a lipophilic cation moiety triphenylphosphonium (TPP+) (Kelso et al., 2001, 2002; National Center for Biotechnology Information, 2019a,b). The positive charge of the TPP+ allows uptake into the cytoplasm and to the mitochondrial membrane via the mitochondrial membrane potential (ΔΨm), where MitoQ has been shown to accumulate to 100–1000 fold over that found in the cytoplasm (James et al., 2007; Smith and Murphy, 2010). MitoQ was initially designed as a mitochondrial specific antioxidant to reduce mitochondrial ROS and block lipid peroxidation, thereby preventing oxidative damage, and improving mitochondrial health (Kelso et al., 2001, 2002). Since its conception, MitoQ has been used in multiple *in vivo* studies across a broad spectrum of diseases; however, a large number of these studies were not performed with an adequate control group, specifically dTPP+. This has resulted in the generation of potentially misleading assumptions regarding the physiological effects of MitoQ. Indeed, it has now been shown in several studies that dTPP+ elicits its own effects, suggesting that many of the beneficial effects previously attributed to MitoQ, may have been driven by the dTPP+ cation. Examples include studies that observed reductions in body weight and fat mass in mice fed a HFD in comparison to mice administered a control agent such as ethanol or cyclodextrin, rather than dTPP+ or dTPP+ complexed with cyclodextrin (Fink et al., 2014, 2017; Imai et al., 2018).

Our current study investigated the metabolic effects of MitoQ on mice fed a HFD, with a focus on WAT biology. These studies were performed in a prevention and regression manner in parallel to mice treated with the same dose of dTPP+.

## Materials and Methods

### Animals

All animal experiments were approved by the Alfred Medical Research and Education Precinct (AMREP) Animal Ethics committee (E/1618/2016/B). Six week old male C57BL/6J mice were sourced from the AMREP Animal Services and housed with up to 6 mice of the same sex per cage. Mice were allowed to acclimatize for 2 weeks prior to feeding of a high fat diet (43% energy from fat, #SF04-001 Specialty Feeds) from 8 weeks of age (prevention studies), or 12 weeks of age (regression studies), for the duration of the study to 18 weeks of age. All mice were housed at 22°C on a 12 h light/dark cycle, had access to food and water *ad libitum* and cages were changed weekly.

Mice were divided into 4 groups: MitoQ1, receiving MitoQ from 8 weeks of age at the commencement of HFD (prevention); dTPP1, receiving dTPP+ from 8 weeks of age at the commencement of HFD (control prevention); MitoQ2, receiving MitoQ from 12 weeks of age after 4 weeks of HFD (regression); dTPP2, receiving dTPP+ from 12 weeks of age after 4 weeks of HFD (control regression) (see Supplementary Figure S1). All groups were randomized by cage and matched to body weight, however prevention and regression groups could not be randomized within the same cage, given that treatments were delivered in a shared water bottle. MitoQ (mitoquinone mesylate) was supplied as a powder (gift from Mike Murphy), which was dissolved in drinking water containing D-glucose (10 mg/ml). Glucose was added to mask any distaste and encourage normal drinking behavior. dTPP+ (Gift from Andrew Murphy at Baker Heart and Diabetes Institute) was prepared, similarly to MitoQ. MitoQ and dTPP+ were made fresh weekly and stored in light proof bottles when in the cages. In order to slowly acclimate mice to treatment, doses of MitoQ and dTPP+ were initially introduced at 100 μM for the first week of treatment, 250 μM for the second week, and 500 μM from the third week onward for both prevention and regression groups. An additional 2 groups were housed under the same conditions on either HFD (*n* = 5) or normal chow diet (*n* = 3) for 10 weeks with no treatment or glucose added to the water as an untreated comparison for respiration and qPCR analysis. At 18 weeks of age mice were humanely euthanized with blood and tissues collected before being snap frozen in liquid nitrogen and stored at −80°C (Supplementary Figure S1).

### EchoMRI

Body composition analysis, including lean mass, fat mass and free water, was measured as previously described (Lancaster and Henstridge, 2018).

### Glucose Tolerance Tests

Oral glucose tolerance tests (oGTT) were performed at three different time points, at a dose of 2 mg/kg of lean mass as determined by EchoMRI (see Supplementary Figure S1). All oGTTs were performed as previously described by Marshall et al. (2018).

### CLAMS

Comprehensive Laboratory Animal Monitoring System (CLAMS, Columbus Instruments, Columbus, OH, United States) was performed as previously described (Lancaster and Henstridge, 2018).

### *Ex vivo* Metabolic Flux Assays

Metabolic flux analyses measuring the oxygen consumption rate (OCR) in *ex vivo* adipose tissue samples were performed using a Seahorse XF24 Extracellular Flux Analyzer (Agilent, Santa Clara, CA, United States). Subcutaneous tissue was excised from mice and immediately placed in Krebs buffer on ice. Approximately 8 mg of subcutaneous adipose tissue was dissected and placed into an islet capture Seahorse plate (Agilent, Santa Clara, CA, United States). A small wire mesh, provided with the islet capture Seahorse plate, was placed on top of the adipose tissue to hold it in place during the assay. Tissue was washed twice with OCR assay media (Seahorse XF base medium supplemented with 25 mM glucose, 2 mM glutamine and 1 mM sodium pyruvate) and then added to each well for a final volume of 500 μl. The plate was then calibrated in a non-CO2 incubator for 10 min before the assay was run. The assay protocol consisted of a calibration and equilibration step followed by repeat cycles of 3 min mix, 2 min wait, and 3 min measurement of OCR. Basal energetics were established after four of these initial cycles followed by sequential injections of the following compounds: the proton ionophore carbonyl cyanide-4-(trifluoromethoxy) phenylhydrazone (FCCP, 8 μM) for 6 measurements, and the mitochondrial complex III and complex I inhibitors antimycin A/rotenone (15 μM) for a further 18 measurements.

### qPCR Analysis

RNA was isolated from tissues using RNAzol reagent and isopropanol precipitation. 1 μg of cDNA was generated from RNA using MMLV reverse transcriptase (Invitrogen) according to the manufacturer’s instructions. qPCR was performed on 10 ng of cDNA using the SYBR-green method on an ABI 7500, using primer sets as outlined in Supplementary Table S1. Quantification of a given gene was expressed by the relative mRNA level compared with control, which was calculated after normalization to a housekeeping gene *cyclophilin A* (*Ppia)* using the delta-CT method. Primers were designed to span exon-exon junctions and were tested for specificity using BLAST (Basic Local Alignment Search Tool; National Centre for Biotechnology Information). Amplification of a single amplicon was estimated from melt curve analysis, ensuring only a single peak and an expected temperature dissociation profile were observed.

### SDS-PAGE and Immunoblot

Tissue was lysed and homogenized in radio-immunoprecipitation assay (RIPA) buffer supplemented with protease and phosphatase inhibitors. Matched protein quantities were separated by SDS-PAGE and transferred to PVDF membranes. Membranes were blocked in 3% skim milk for 2 h and then incubated with primary antibody overnight at 4°C. After incubation, membranes were probed with a HRP-conjugated secondary anti-mouse/rabbit antibody in 3% skim milk for 2 h at room temperature, then visualized with chemiluminescence (Pierce). Approximated molecular weights of proteins were determined from a co-resolved molecular weight standard (BioRad, #1610374). OXPHOS (MitoSciences), 4HNE (Abcam), pan 14-3-3 (Santa Cruz).

### Statistical Analysis

All data are expressed as mean ± standard error of the mean (SEM). All statistical analyses was performed using PRISM7 software. Results for *in vivo* studies were analyzed by one or two-way repeated measures analysis of variance (RM-ANOVA), or paired Student’s *t*-test where appropriate.

## Results

### MitoQ and dTPP+ Reduce Food and Water Intake and Mitigate Weight Gain on a HFD

Given several studies have documented significant effects of MitoQ treatment on indices of adiposity in diet induced obesity (DIO) models, we sought to determine if these effects were dependent, or independent of the dTPP+ cation. To investigate the effects of MitoQ on weight gain and adiposity, we administered MitoQ and dTPP+ (500 μM) to mice at the commencement of a HFD (prevention group, MitoQ1 or dTTP1), and to a separate cohort of mice that had already been fed a HFD for 4 weeks (regression group MitoQ2 or dTPP2 – see Supplementary Figure S1). It has been demonstrated previously that MitoQ at a concentration of 500 μM in the drinking water is sufficient to elicit effects in mice without toxicity (Smith and Murphy, 2010). All groups had D-glucose added to their water from the commencement of the HFD in an attempt to mask the taste of the dTPP+ and MitoQ, and to encourage normal drinking habits.

**FIGURE 2:**
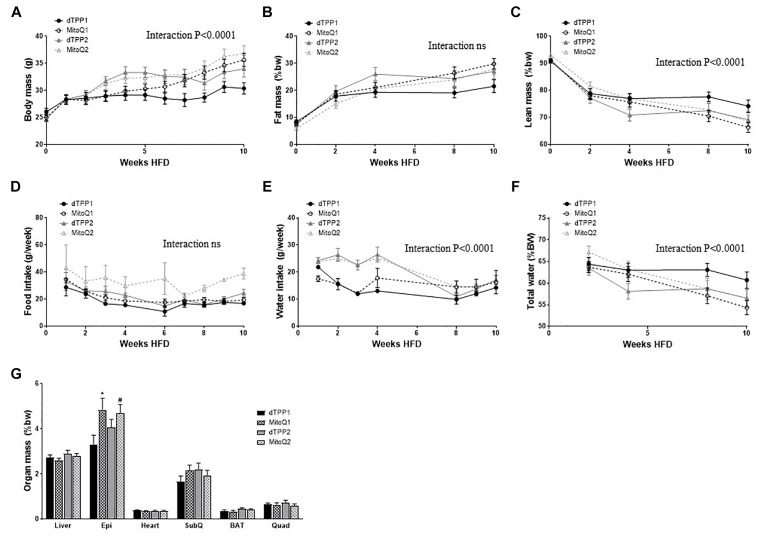
Mitochondrial-targeted coenzyme Q and (1-Decyl)triphenylphosphonium bromide prevent weight gain via a reduction in food and water intake. Body composition of mice fed a HFD in the prevention group (dTPP1 and MQ1 – Black) and regression group (dTPP2 and MQ2 – Gray). **(A)** Body mass; **(B)** fat mass; **(C)** lean mass; **(D)** food intake; **(E)** water intake (g/week); **(F)** water intake (%BW) over time and **(G)** organ mass as percent body weight from mice after 10 weeks of HFD. Epi, epididymal fat; SubQ, subcutaneous fat; BAT, brown adipose tissue; and Quad, quadricep muscle. Values represent mean ± SEM (9 mice/group). ^∗^*p* < 0.05 from dTPP1 and dTPP2, #*p* < 0.05 from dTPP1.

Mice administered MitoQ and dTPP+ from the commencement of HFD (prevention group – Black lines) demonstrated a significant (interaction of time × treatment *p* < 0.0001), yet equivalent protection against weight gain for the first 4 weeks of HFD, compared with the regression group – which had yet to be administered any treatment (MitoQ2 and dTPP2 – Gray lines) (Figure 2A). After 5 weeks, when all groups were receiving treatment, there was a distinct plateau in the weight gain in the regression groups (Figure 2A). The differences in weight between these groups mostly persisted for the remainder of the study, however the MitoQ1 group showed a significant gain in weight compared with dTPP1. This is consistent with previously published data, however we show that the effects from treating mice with dTPP+ are not significantly different to those seen with MitoQ. Furthermore, we observe that there are no significant differences in fat mass or lean mass determined by EchoMRI between the MitoQ and dTPP+ treatment groups, however there was an overall significant (*p* < 0.0001) decline in lean mass within the groups (Figure 2B,C). These data suggest that there are no differences in these parameters between MitoQ and dTPP+ treatments.

Although the reductions in weight gain could be perceived as being beneficial, these reductions may be attributed to reduced food intake (not significant as time × treatment interaction) or water intake (time × treatment interaction; *p* < 0.0001) (Figure 2D,E). The initial decline in food intake is likely due to the switch of diet from CHOW to HFD, however this stabilized for all groups from 4 to 6 weeks after commencement of HFD (Figure 2D). Water intake in the first few weeks was significantly (*p* < 0.0001) lower in the prevention groups compared with the regression groups (yet to receive treatment), which notably declines in the regression group once they commenced treatment after 4 weeks of HFD (Figure 2E). The reduction in water intake in the regression group coincides with the reduction in weight gain shown in Figure 2A, suggesting that reductions in weight gain can be largely attributed to reductions in water intake. Nevertheless, to ensure that the mice were not dehydrated, we measured total free water using EchoMRI and observed that even in the setting of reduced water intake, the mice were not significantly lower in whole body free water content (Figure 2F).

With regards to organ weights, we show a significant (*p* < 0.05) increase in epididymal fat pad mass in both MitoQ treated groups, compared with the dTPP1 group (Figure 2G). We also observed that the dTPP1 group likely had reduced caloric intake as a result of consuming less food and water than MitoQ1, MitoQ2, and dTPP2 groups (Figure 2D,E). No significant changes were observed in organ mass in any other tissues (Figure 2G).

Collectively, these results suggest that the reduction in weight gain in mice treated with MitoQ and fed a HFD is likely due to the dTPP+ cation and intolerance of the treatments, which manifested as a reduction in water intake, rather than an antioxidant specific effect of MitoQ. We also observed that MitoQ and dTPP+ were not well tolerated by the mice, as indicated by reduced water and food intake during treatment periods.

### MitoQ Does Not Improve Glucose Tolerance in High Fat Fed Mice Compared With dTPP+

**FIGURE 3:**
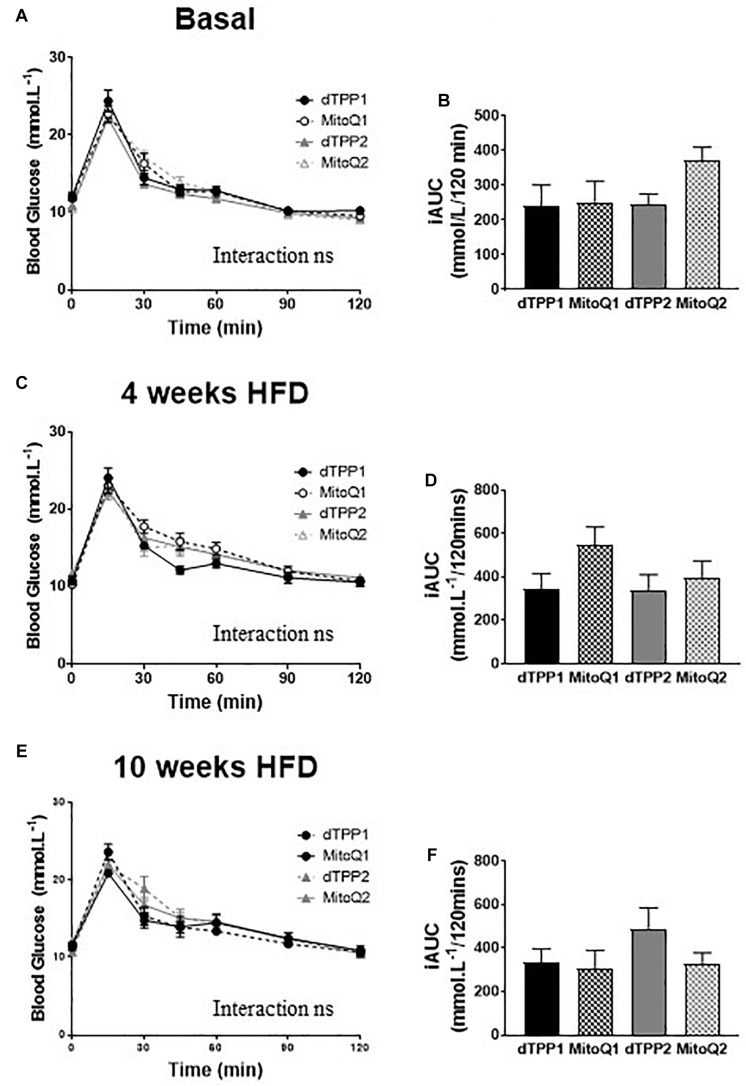
Mitochondrial-targeted coenzyme Q and (1-Decyl)triphenylphosphonium bromide have no beneficial or adverse effect on glucose tolerance. Oral glucose tolerance tests in prevention (dTPP1 and MQ1 – Black) and regression (dTPP2 and MQ2 – Gray) groups with incremental area under the curve at the commencement of HFD **(A**,**B)**, and 4 weeks (**C**,**D**, before regression group treatment), or 10 weeks **(E**,**F)** after commencement of HFD. Values represent mean ± SEM (9 mice/group).

To determine whether MitoQ and dTPP+ had effects on glucose tolerance, we performed oral glucose tolerance tests (oGTTs, 2 mg/kg lean mass) prior to the commencement of HFD (Figure 3A,B), after 4 weeks of HFD (before regression group commenced treatment, Figure 3C,D), and after 10 weeks of HFD (Figure 3E,F). We observed that there were no significant differences in glucose tolerance between any of the groups after 0, 4, or 10 weeks of HFD, demonstrating that there is no effect of MitoQ on glucose tolerance in DIO mice compared to dTPP+ treatment. This was further highlighted by the lack of significant difference in incremental area under the curve (iAUC; change from baseline fasting blood glucose) between all groups for oGTTs at all time points (Figure 3B,D,F). Notably, although 10 weeks of HFD is usually sufficient to impair glucose tolerance, we detected no impairment in glucose tolerance for all treatment groups on a HFD (Figure 3E). Thus, when compared with glucose intolerance normally observed in mice fed a HFD diet for 10 weeks (with no MitoQ or dTPP+ treatment), our data are in agreement with previous findings that MitoQ treatment had a positive effects on glucose tolerance; however, similar findings were observed for dTPP+. These results suggest that if there are beneficial effects of MitoQ and dTPP+ on glucose tolerance they are likely due to weight loss and reductions in fat mass, and not specifically related to the antioxidant moiety of MitoQ.

### MitoQ and dTPP+ Increase Resting Energy Expenditure Compared With Untreated Mice

**FIGURE 4:**
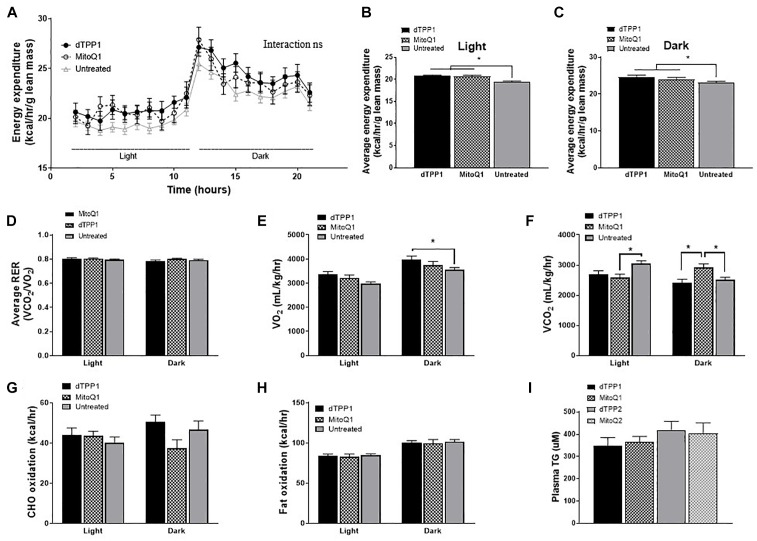
Mitochondrial-targeted coenzyme Q and (1-Decyl)triphenylphosphonium bromide increase energy expenditure. Data from metabolic cages after 4 weeks of HFD and 4 weeks of dTPP+/MitoQ (prevention group; dTPP1 and MQ1 – Black) or 0 weeks of dTPP+/MitoQ (untreated – Gray). **(A)** Energy expenditure over 24 h; light, rest; dark, active periods. Average energy expenditure during the **(B)** light period and **(C)** dark period; **(D)** average respiratory exchange ratio (RER) during the light and dark period; **(E)** oxygen consumption and **(F)** carbon dioxide production during the light and dark periods; **(G)** carbohydrate oxidation (CHO) and **(H)** fat oxidation during the light and dark periods; **(I)** plasma triglycerides levels after 10 weeks of HFD. Values represent mean ± SEM (dTPP1 = 9 mice, MitoQ1 = 9 mice, andUntreated = 18 mice), ^∗^*P* < 0.05.

There is some conjecture as to whether metabolic changes induced by the dTPP+ cation are a result of changes to the ΔΨm, proton leak, uncoupled respiration, or a combination of all three. Increased proton leak and uncoupled respiration will likely lead to increased thermogenesis resulting in increased energy expenditure and heat production. Thus, in order to determine whether MitoQ and dTPP+ altered energy expenditure and heat production, we placed all 4 groups of mice into metabolic cages after 4 weeks of HFD, half of which had been receiving MitoQ/dTPP+ for 4 weeks (prevention groups), and half which had not (both regression groups combined – annotated as non-treated). We demonstrated that treatment with either dTPP+ or MitoQ (black lines) for 4 weeks resulted in an upward shift in energy expenditure (normalized to lean mass) in both light and dark periods (Figure 4A), suggesting that treatment with MitoQ and dTPP+ for 4 weeks was likely inducing either mitochondrial proton leak or uncoupled respiration. When averaging these data over the light and dark periods there was a significant (*p* < 0.05) increase in energy expenditure in both treatment groups at rest (light) and during the active period (dark) compared with the non-treated group (Figure 4B,C).

Although there were no significant changes in RER between the groups (Figure 4D), we did observe a trend for increased O_2_ consumption (VO_2_) and significant decreases in CO_2_ production (VCO_2_) in the 4 week treatment groups during rest (light period; Figure 4E,F), which may also indicate alterations in the number of uncoupled mitochondria. Lastly, we observed significantly higher CO_2_ production in the MitoQ1 group compared with non-treated and dTPP1 groups during the dark (active) period (Figure 4F).

Using data from the CLAMS analysis, we were able to calculate carbohydrate (CHO) and lipid oxidation rates in mice to estimate whether substrate switching might account for the observed changes in energy expenditure. We detected substantial variability in CHO oxidation during the active period (dark period), although these were not significant (Figure 4G). We also demonstrated no change in fat oxidation between groups in light or dark periods (Figure 4H). Finally, plasma triglycerides measured at the end of the study suggested a trend for reduced triglycerides in the prevention groups (MitoQ1 and dTPP1) compared with the regression groups (Figure 4I). This is consistent with other studies that demonstrated that MitoQ can reduce triglyceride levels (Rodriguez-Cuenca et al., 2010); however, our data suggest that this is likely a reflection of MitoQ and dTPP+ on reduced body weight and fat mass, and not due to the antioxidant activity from MitoQ as previously suggested.

Overall, these metabolic data suggest that binding of dTPP+ to the mitochondrial membrane alters mitochondrial respiration, resulting in increased energy expenditure. These data further suggest that treatment with MitoQ in the current studies, does not exert effects through the antioxidant moiety, but rather through general effects elicited by the dTPP+ cation.

### MitoQ and dTPP+ Reduce Mitochondrial Respiration in Adipose Tissue

**FIGURE 5:**
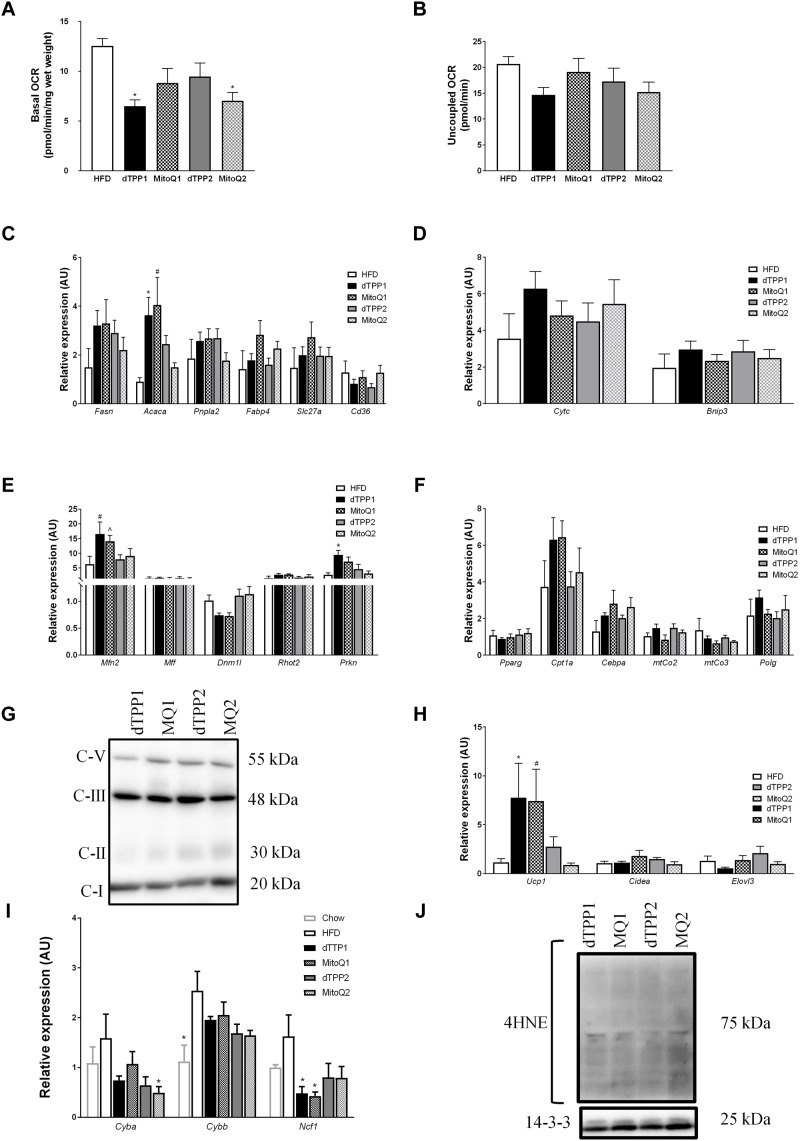
Mitochondrial-targeted coenzyme Q and (1-Decyl)triphenylphosphonium bromide reduce mitochondrial respiration and alter gene expression. **(A)** *Ex vivo* basal and **(B)** uncoupled oxygen consumption in epididymal fat after 10 weeks of HFD, ^∗^*p* < 0.05 vs. HFD. mRNA expression in epididymal fat pads for **(C)** metabolic (^∗^*p* < 0.05 vs. MQ2, HFD; #*p* < 0.05 vs. MQ2, HFD), **(D)** apoptotic, **(E)** mitochondrial dynamic (#*p* < 0.05 vs. dTPP2, MQ2, HFD; ˆ*p* < 0.05 vs. dTPP2, HFD; ^∗^*p* < 0.05 vs. MQ2, HFD), and **(F)** adipogenic and mitochondrial genes. **(G)** Immunoblot for OXPHOS protein complexes including complex 1 (C-I), complex 2 (C-II), complex 3 (C-III), and complex 5 (C-V). **(H)** mRNA expression from epididymal fat for genes associated with adipose tissue browning (^∗^*p* < 0.05 vs. dTPP2, MQ2; #*p* < 0.05 vs. MQ2), and **(I)** genetic markers of oxidative stress (^∗^*p* < 0.05 vs. HFD). **(J)** Immunoblot for 4-hydroxynonenal (4HNE) and loading control 14.3.3. Values represent mean ± SEM; Treated groups (*n* = 9 mice/group) and untreated (*n* = 5 mice).

To test whether MitoQ and the dTPP+ can directly affect WAT mitochondrial respiration, either through changes to the ΔΨm, proton leak, or uncoupled respiration, we used the Seahorse XF bioanalyzer to measure bioenergetics in adipose tissue *ex vivo*. To do this, we excised fresh subcutaneous adipose tissue samples from treated mice at the conclusion of the study, and immediately measured their respiratory capacity. Representative traces for these experiments can be found in Supplementary Figure S2. As a comparison for these studies, we used a separate cohort of mice fed HFD for 10 weeks in the absence of treatment with MitoQ or dTPP+. These mice were not used in the study for comparisons in measures of obesity or glucose tolerance. We demonstrated that there was no difference in either basal or uncoupled respiration in WAT between MitoQ/TPP+ groups (Figure 5A,B), however we did demonstrate that dTPP+ and MitoQ reduced basal mitochondrial respiration in WAT compared with untreated HFD fed mice (Figure 5A). These results may reflect the state of mitochondria in tissue from mice treated with MitoQ or dTPP+, where reduced mitochondrial respiration could be due to mitochondrial membrane depolarization.

To further determine changes in metabolic parameters in WAT, we measured gene expression in epididymal fat pads from HFD fed mice treated with or without MitoQ or dTPP+. Consistent with the trend observed for reduced plasma triglyceride levels in the prevention group shown above (Figure 4I), we demonstrated increased mRNA expression of *Acaca* in the prevention group compared with the regression group (Figure 5C). *Acaca* encodes the protein acetyl-CoA carboxylase, which regulates the metabolism of fatty acids through its actions either in the mitochondria or the ER to inhibit beta oxidation or activate lipid biosynthesis, respectively. This suggests that mice treated with MitoQ and dTPP+ are favoring lipid storage and uptake, which may reflect low reserves of adipose tissue, and reduced food intake. Although we observed no significant changes in other metabolic genes including *Pnpla2* and *Slc27a*, we observed trends for increased expression of *Fasn* in all groups compared to HFD with no treatment, and in *Fabp4* and *Cd36* in MitoQ treated groups compared to dTPP+ groups (Figure 5C).

Next we wanted to confirm whether the observed reductions in respiration were due to apoptosis or other pathways relating to cellular damage via the assessment of the key apoptotic markers *Cytc* and *Bnip3*. We demonstrated no significant difference between all groups, suggesting that apoptosis was unlikely to explain the reduced respiration (Figure 5D). Subsequently, we assessed the expression of genes involved in mitochondrial dynamics and demonstrated that the mitochondrial fusion gene, *Mfn2* was increased in the prevention groups compared with the untreated HFD group (Figure 5E). In addition, the expression of *Mfn2* is significantly higher in the prevention groups compared with the regression groups, which coincides with a trend for reduced expression of the mitochondrial fission gene *Dnm1l* (Figure 5E). These findings suggest a potential increase in mitochondrial fusion in the prevention and regression groups compared with the untreated groups, which is more evident in the longer term (prevention) treated group. This could be explained by the continued steady-state accumulation of dTPP+ in the mitochondria, where previous studies have demonstrated that dTPP+ accumulates over time within the mitochondrial membrane up to 1000-fold (Smith and Murphy, 2010; Trnka et al., 2015). Increased mitochondrial fusion could be a compensatory response for changes in ΔΨm that lead to proton leak and uncoupled respiration. This is consistent with the observed increase in *Prkn* expression in the treatment groups, compared with the untreated mice/group, indicating that that there may be an increased signal to remove unhealthy mitochondria via mitophagy (Figure 5E). We did not detect any changes in mRNA expression of the mitochondrial fission protein, *Mff* or the mitochondrial trafficking protein, *Rhot2* (Figure 5E).

Finally, we investigated other key mitochondrial and adipogenic genes to determine whether there were any changes in mitochondrial number, adipose expansion or browning of WAT. We observed no significant changes between all treatment groups or in comparison to the untreated group with regard to key mitochondrial genes including mitochondrial cytochrome c oxidase II or III, (*mtCo2*, *mtCo3*), or polymerase gamma (*Polg*) suggesting that there were no changes in mitochondrial number in WAT (Figure 5F). In support of this, we immunoblotted for ETC complex proteins in WAT and observed no significant differences in protein abundance (Figure 5G). In addition, as a measure of adipose tissue exapnasion we measured expression of the master regulator of adipogenesis, *Pparg* and observed no changes between all groups (Figure 5F). To determine whether adipocyte browning occurred, which could also account for increased energy expenditure and reduced fat mass, we measured key genes involved in the activation of brown adipose. We observed a significant increase in *Ucp1* mRNA expression in the prevention groups, but no differences in other markers of adipose browning such as *Cidea* and *Elovl3*, suggesting that UCP1 may be increased in relation to uncoupled mitochondrial respiration, rather than browning/beiging of the adipose (Figure 5H). Furthermore, we demonstrate using both molecular and protein measures of oxidative stress that both dTPP+ and MitoQ have minimal effect to reduce oxidative stress, and in fact any reductions in oxidative stress may be due to inhibited ETC function and reduced respiration (Figure 5I,J). These data support other findings that the dTPP+ cation induces changes in either ΔΨm, uncoupled respiration or proton leak, and inhibits the ETC and oxidative phosphorylation (Trnka et al., 2015; Gottwald et al., 2018).

Collectively, our results demonstrate that the previously documented effects of MitoQ in DIO mice are likely due to the dTPP+ cation and not due to the antioxidant effects of the MitoQ moiety. Furthermore, our results also indicate that there is no overt metabolic benefit in the adipose with MitoQ treatment at the dose regimen used in our study. These results highlight the need for the appropriate control, dTPP+, to be used in future studies that utilize MitoQ, and also demonstrate that dosing regimens should be titrated for specific outcomes to maximize tolerability in mice.

## Discussion

As the prevalence of obesity continues to rise worldwide it is becoming increasingly important to develop new therapeutics to treat subsequent complications such as MS and type 2 diabetes. There is a well demonstrated effect of antioxidants on signaling properties of ROS within cells, which in turn, regulates many cellular processes (Hirzel et al., 2013). However, to date there has been little success with antioxidants as a therapeutic intervention for metabolic disease, possibly related to the inability to deliver sufficient doses of antioxidant to the required site. With the development of MitoQ, a highly cell penetrable compound with specific affinity for mitochondria, the ability to deliver antioxidants specifically to the mitochondria *in vivo* became possible. Indeed, several studies have investigated the potential benefits of MitoQ in mice fed a HFD. These studies have established that MitoQ treatment can prevent weight gain in the setting of HFD, however we show that these effects are likely due to the dTPP+ cation, rather than the antioxidant effects exerted by ubiquinone *per se*. Some studies that have performed co-treatment of mice with dTPP+ as a control, did not present data on body weight or that relating to food, and water intake (Rodriguez-Cuenca et al., 2010; Rehman et al., 2016). However, others have indeed presented dTPP+ data, which confirmed similar results to those presented here in our study. Other groups have further suggested that MitoQ may even be a cytotoxic compound that induces a collapse of the ΔΨm, which significantly reduces mitochondrial ATP production and oxygen consumption (Alshamrani et al., 2016).

Our results indicate that MitoQ has no added benefit in mice on a HFD compared to dTPP+, and although we observe a reduction in weight and fat mass, this can be mostly attributed to reduced water and caloric intake. These effects could be a result of an accumulation of the dTPP+ cation in the mitochondrial membrane, rather than any antioxidant effects from ubiquinone. Specifically, we demonstrated that there was no independent beneficial effect of MitoQ on glucose tolerance in mice fed a HFD for 10 weeks. Another consideration in interpreting these results is the apparent low tolerance that mice have to these compounds.

Mitochondrial-targeted coenzyme Q has been shown to steadily accumulate in the mitochondria, where even micro-molar concentrations can disrupt membrane integrity leading to mitochondrial dysfunction; however, accumulation at nano-molar concentrations is suggested to be protective for membrane integrity (Cocheme et al., 2007). This speaks to the difficulty of achieving the correct dose of MitoQ in different tissues that vary greatly in their mitochondrial density, and with a compound that steadily accumulates in the mitochondria over time. Such complexities confound the administration of MitoQ and other dTPP+ compounds as interventions to reduce ROS and treat mitochondrial dysfunction. Reily et al. (2013) conclude that the concentrations of dTPP+ required as a delivery system would be difficult to achieve, given that dTPP+ depends on plasma and ΔΨm, cell volume, and the number of mitochondria within a cell.

There is some conjecture over how dTPP+ and MitoQ exert their effects, and our results and others indicate that there are metabolic alterations occurring as a result of administering these compounds. Reily et al. (2013) demonstrated that various dTPP+ compounds inhibited oxidative phosphorylation to differing extents, independent of the antioxidant functional groups, and instead demonstrated that alterations to mitochondrial function, accumulation, and retention are dependent on the alkyl chain length of the linker. It has also been suggested that MitoQ may lead to mitochondrial uncoupling or a reduction in ΔΨm (Trnka et al., 2015). Pertinent to the current study, Hirzel et al. (2013) showed a reduction in basal oxygen consumption in human adipocytes treated with MitoQ and dTPP+, suggesting that the dTPP+ cation inhibits mitochondrial respiration in adipocytes, which could be due to mitochondrial membrane depolarization. We observed few changes in WAT respiration between the MitoQ and dTPP+ groups from excised subcutaneous fat pads, suggesting that there is no effect from the quinone moiety; however, we did demonstrate that both groups had reduced respiration compared with HFD controls in the absence of dTPP+ compounds, suggesting that dTPP+ inhibits oxidative phosphorylation. Furthermore, our data shows no difference between oxidative stress between dTPP+ and MitoQ, which could be related to inhibition of the ETC with these compounds.

We demonstrated changes in key mitochondrial and metabolic genes, however these were not due to the antioxidant moiety, as changes were seen in both MitoQ and dTPP+ groups. However, we did observe differences between the prevention and regression groups. This might be explained by a gradual accumulation of dTPP+ cations on the mitochondrial membrane, resulting in alterations in gene expression. We demonstrated that there was an increase in expression of the mitochondrial fusion gene *Mfn2* and the metabolic gene *Acaca*, which suggests changes to mitochondrial fusion programs and energy utilization. Because mice treated with dTPP+ or MitoQ were shown to have reduced weight and fat gain, the nutrient preservation pathways would have been activated to favor energy storage resulting in an upregulation of genes involved in mitochondrial fusion and lipid synthesis in WAT. These data are consistent with previous results, suggesting that MitoQ does not have independent beneficial effects over dTPP+ (Trnka et al., 2015; Gottwald et al., 2018).

In summary, our results support the notion that the observed effects with MitoQ treatment on bioenergetics in mice are most likely due to the steady state accumulation, and non-specific effects of the dTPP+ cation, rather than the MitoQ antioxidant moiety. We noted no significant changes in oxidative stress or energy metabolism in adipose tissue as a direct result of MitoQ treatment *per se*. We also observed that the animals had a poor tolerance to the dTPP+/MitoQ treatment, which suggests that achieving an optimum dosage whilst balancing toxicity will present a significant hurdle in any therapeutic applications. In conclusion, future studies should take into consideration the non-specific effects of dTPP+ and use the appropriate controls when investigating dTPP+ compounds such as MitoQ.

## Author Contributions

BD and SB designed and conceived the study and wrote the manuscript. SB and JK performed all the experiments. AC provided reagents, experimental advice, and access to resources. All authors read and edited the manuscript.

## Conflict of Interest Statement

The authors declare that the research was conducted in the absence of any commercial or financial relationships that could be construed as a potential conflict of interest.
