# Supplementary figures and images for: The Antioxidant Moiety of MitoQ Imparts Minimal Metabolic Effects in Adipose Tissue of High Fat Fed Mice
Source: Front Physiol. 2019 May 8;10:543. doi: 10.3389/fphys.2019.00543 (PMC6517842; doi:10.3389/fphys.2019.00543)

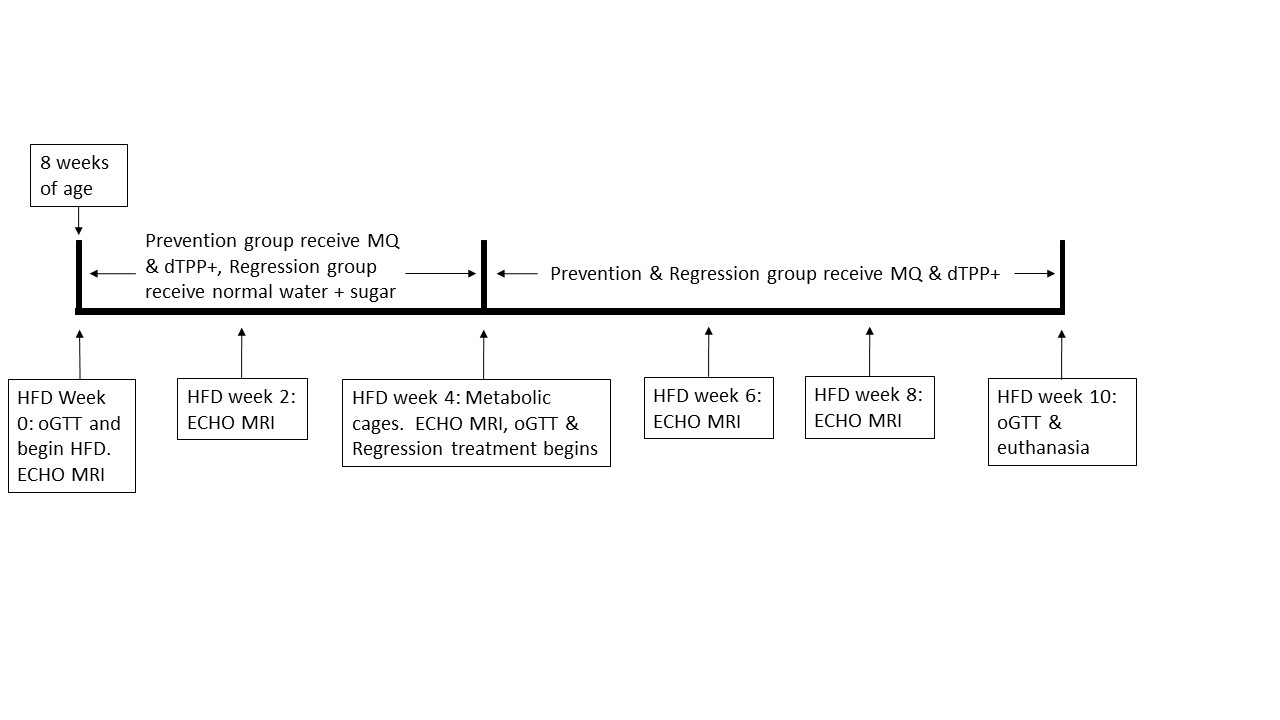

Supplement: Supplementary file 1 [file Image_1.JPEG]

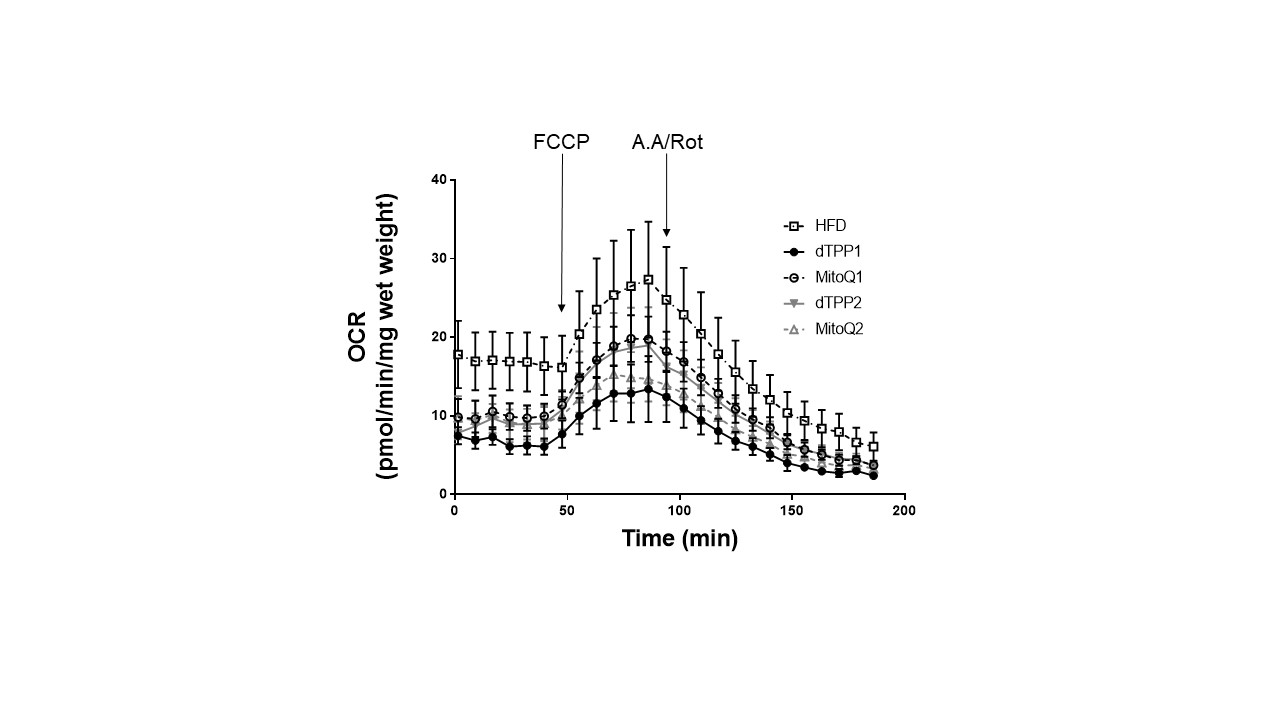

Supplement: Supplementary file 2 [file Image_2.JPEG]

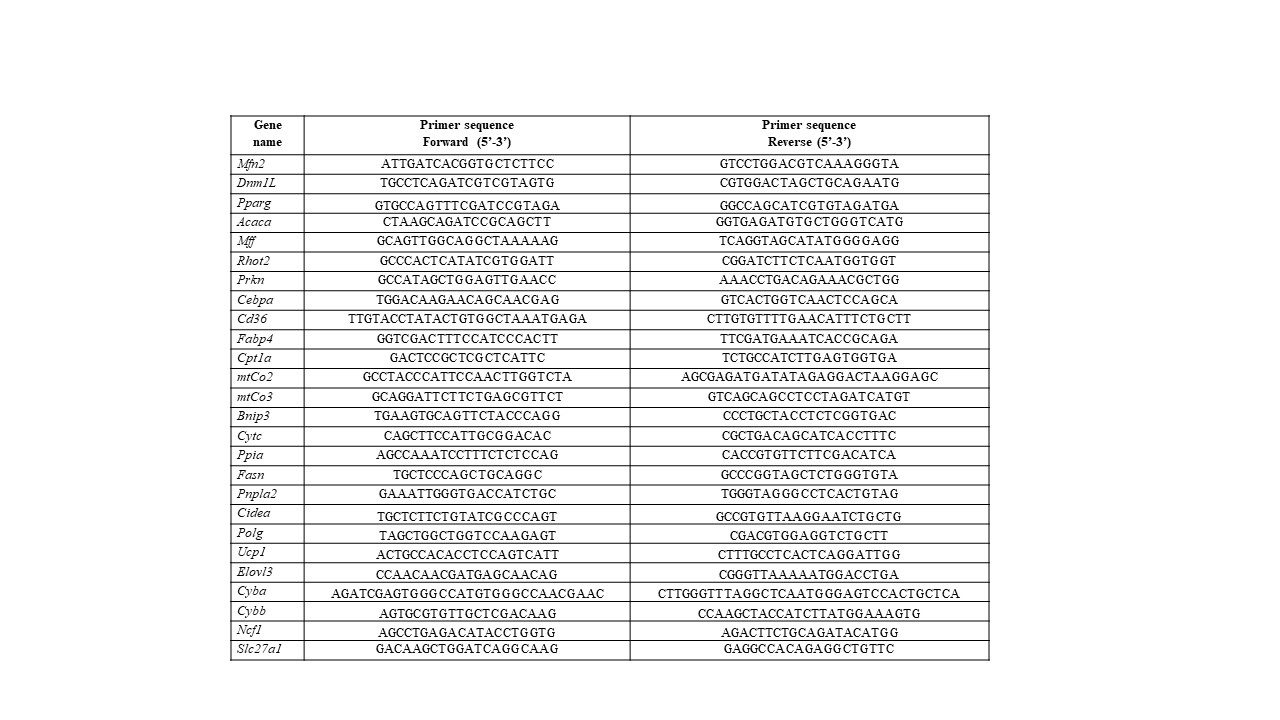

Supplement: Supplementary file 3 [file Image_3.JPEG]
